# Staining of Tubercle Bacilli in Tissue Preserved in Alcohol for Sixty Years

**Published:** 1908-09

**Authors:** A. A. Thibaudeau

**Affiliations:** Pathological Laboratory, University of Buffalo


### Staining of Tubercle Bacilli in Tissue Preserved in Alcohol for Sixty Years.

By A. A. THIBAUDEAU, M. B.,
Pathological Laboratory, University of Buffalo.

MOST observers believe that the bacillus of tuberculosis will retain its peculiar staining properties for a long time. A hasty review of some of the literature and more particularly of the various numbers of Baumgartens Jahresbericht has, however, failed to reveal any observations on the length of time that this organism will stain in the usual characteristic fashion in hardened tissues. On this account, some results obtained in the Pathological Laboratory of the University of Buffalo may be of interest.

The tissues were from specimens of tuberculosis of the lung donated to the pathological museum of the University of Buffalo by the late Austin Flint, then professor of the principles and practice of medicine, in 1848—just sixty years ago. During the past sixty years, these specimens have been preserved in alcohols of varying strengths. They include three specimens of acute tuberculosis with miliary tubercles and tuberculous pneumonia and one of chronic fibrous tuberculosis. Sections of these specimens showed the usual histology but they took the hematoxylin and eosin stain somewhat more feebly than would have been expected of ordinary, well preserved tissue.

Smears were made from suitable portions of all four specimens and stained with carbol-fuchsin for a few minutes using occasional steaming. In decolorising a weak solution (1|3 per cent.) of $HC_1$ in 70 per cent. alcohol was used. This rapidly removed the color from the smears, which were then washed in water and counterstained. By this method typical tubercle bacilli were demonstrated in the three cases of acute tuberculosis. None were found in the smears from the case of chronic fibrous tuberculosis.

Sections of tissue from these cases were also stained in carbol-fuchsin. It was found advisable to expose the sections for a long time to the action of the warmed stain. In decolorising the same weak solution (1|3 per cent.) of $HC_1$ in 70 per cent. alcohol was used very briefly. Sections were transferred from this solution to alcohol, cleared in xylol and mounted. By exercising some care in the staining and more particularly in decolorising, in the sections as in the smears, tubercle bacilli were demonstrated in the cases of acute tuberculosis, while they were not found in the case of the chronic fibrous tuberculosis. It is proper to add, however, that in many sections of the first three cases no tubercle bacilli at all could be demonstrated, while in other sections the bacilli were few in number and were feebly and irregularly stained. In only a few sections were fairly numerous and typically stained bacilli secured.

From these experiments in the staining of the bacillus of tuberculosis we can state that this organism, when present in tissues preserved in alcohol, may retain for a considerable time—in our case for sixty years—its peculiar staining properties. The staining properties of the bacillus appeared, however, to be somewhat impaired.

---

To prevent unsightly scars, incisions should be made on the slant rather than at right angles to the surface.—*International Jour. Surgery.*

